# Wine Yeast Terroir: Separating the Wheat from the Chaff—for an Open Debate

**DOI:** 10.3390/microorganisms8050787

**Published:** 2020-05-25

**Authors:** Hervé Alexandre

**Affiliations:** UMR Procédés Alimentaires et Microbiologiques, Université de Bourgogne Franche-Comté/AgroSup Dijon, Equipe VAlMiS (Vin, Aliment, Microbiologie, Stress), Institut Universitaire de la Vigne et du Vin Jules Guyot, rue Claude Ladrey, BP 27877, 21000 Dijon, France; rvalex@u-bourgogne.fr

**Keywords:** yeast, terroir, next-generation sequencing, grape vine, alcoholic fermentation, microbial terroir, yeast biodiversity

## Abstract

Wine terroir is characterized by a specific taste and style influenced by the cultivar of the fermented grapes, geographical factors such as the vineyard, mesoclimate, topoclimate, and microclimate, soil geology and pedology, and the agronomic approach used. These characteristics together define the concept of “terroir”. Thus, regional distinctive flavors in wine have been the subject of many studies aimed at better understanding the link between the wine and the vineyard. Indeed, the identification of key environmental elements involved in the regional variation of grape and wine quality characteristics is a critical feature for improving wine production in terms of consumer preference and economic appreciation. Many studies have demonstrated the role of abiotic factors in grape composition and consequently in wine style. Biotic factors are also involved such as grape microbial communities. However, the occurrence and effects of region-specific microbiota in defining wine characteristics are more controversial issues. Indeed, several studies using high throughput sequencing technologies have made it possible to describe microbial communities and revealed a link between grape must and soil microbial communities, and the geography of the territory. Based on these observations, the concept of “microbial terroir” emerged. However, this concept has been subject to contradictory studies. The aim of this opinion article is to take a step back and examine in perspective the concept of microbial terroir, by comparing numerous data from different studies and providing arguments in favor of or against this concept to stimulate discussion and point out that experimental research is still needed to study the contribution of this assembly of microorganisms to the final product and to support or refute the concept.

## 1. Introduction

**Figure 1:**
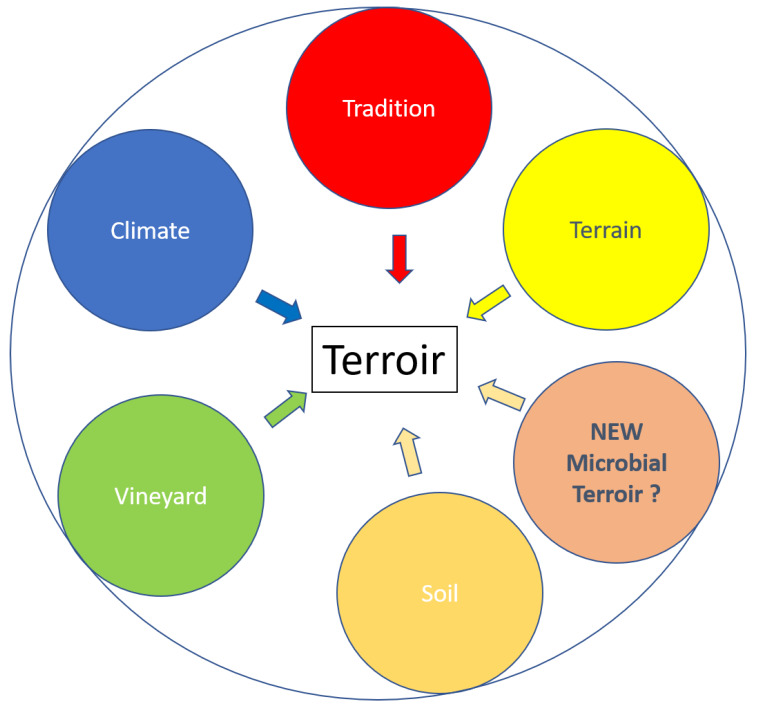
the components of wine terroir.

Since more than 30 years ago, much work has been carried out on the ecology of wine yeasts [1], due to the use of genetic methods for yeast strain identification. Based on different studies, it appears that the *Saccharomyces cerevisiae* (*S. cerevisiae*) strain could be representative of an enological area, namely “terroir” [2]. More recently, microbial aspects have been proposed as participating in the terroir concept that reflects geographical origin (Figure 1). This has been possible because of recent advances in high-throughput, short-amplicon sequencing (HTS) technologies, which have revolutionized the study of microbial communities [3]. Different reports based on yeast ecological studies point to a possible linkage between grape yeast and wine typicality [4,5]. Thus, according to these studies, different viticultural terroirs harbor distinctive yeast biota. Indeed, yeast biodiversity in vineyards is affected by grapevine cultivar macro- and microclimatic conditions and the geographical location of the vineyard, which could explain that yeast consortia between two different wine regions are different. Furthermore, regarding *S. cerevisiae*, which plays a major role in alcoholic fermentation, different studies based on metapopulation analysis have demonstrated the existence of a biogeographical distribution of *S. cerevisiae*, which again points to the existence of typical *S. cerevisiae* strains [4].

However, several elements contradict the terroir concept. Most studies do not take into account that yeast species distribution is subject to significant intra-vineyard spatial fluctuations which may, in part, account for differences between regional yeast microbiota [6]. Another intriguing aspect of the yeast terroir concept is the fact that alcoholic fermentation is mainly conducted with *S. cerevisiae*; in these conditions, what role do the other species play in the typicality of wines? In other words, is *S. cerevisiae* solely responsible for such reported typicality? Recent studies have demonstrated that natural fermentation is conducted with a consortium of yeast species present in the must (grape juice) and which evolve during the alcoholic fermentation [7]. Yeast population dynamics during alcoholic fermentation is complex and depends on environmental factors (must characteristics) and interactions between microorganisms, since it is known that these interactions are strain dependent [7]. This means that whatever the grape microbiota, the yeast dynamics profile will be shaped by many different factors such as yeast fitness, yeast competition, anthropogenic factors, etc. Moreover, the wine yeast terroir concept is based on the assumption that wines are produced by spontaneous fermentation carried out by the yeast biota naturally present in musts (grape juice). Indeed, this is true but the yeast biota in must is a mix of different yeast species present both on the grapes and in the winery (equipment, tanks, and so forth) [8]. Different reports have shown that *S. cerevisiae* and other yeast species colonize the cellar and were found to be responsible for alcoholic fermentation, which is called the winery effect [9,10]. Population analysis performed using Fst genetic distance or ancestry profiles revealed exchanges of *S. cerevisiae* between wine estates [11]. All these data support the view that whatever the origin of the grapes, once in the cellar, it will be colonized by yeast species present in the cellar that will ferment together with the grape consortia or displace it. These latter studies do not support the view that grape yeast biodiversity is well correlated to specific terroirs. Indeed, whether or not a wine terroir includes a microbial aspect has so far not been confirmed. Many studies have shown the existence of regional yeast species microbiota; however, the notion of terroir is not confined only to different regions, as different terroirs can exist within a very narrow region, for example, Burgundy [12]. Two different terroirs can be separated by a few meters and although their respective wines are different, one may ask whether they share the same microbiota. To answer this question, we need to know how the microbial community is built in these terroirs. Where do the microbes come from, since grapes are only present during a few months a year? From the soil? From wasps? Interestingly, it has been shown that social wasps are a key environmental niche for the evolution of natural *S. cerevisiae* populations and the dispersion of yeast cells in the environment [13]. This means that the dispersion of yeast cells by wasps could occur in different closed terroirs and that the same strain could be found in these different terroirs.

The aim of this opinion article is to examine the concept of microbial terroir in perspective by comparing numerous data from different studies, and to provide arguments in favor of or against this concept so as to stimulate discussion and point out that experimental research is still needed to study the contribution of this assembly of microorganisms to the final product and to support or refute the concept.

## 2. Is *S. cerevisiae* Part of the Terroir Effect?

In viticulture, the terroir effect is a complex concept and can be explained by agronomic interactions between the vine and its environment, including human intervention through viticulture and enological practices. The concept of terroir has been defined by the International Organisation of Vine and Wine (OIV) (2010). Although consensual among the members of the OIV, there are some controversies [14]. However, wines with the distinctive characteristics of a particular area are the consequence of the “terroir effect”. Likewise, since yeasts are partly responsible for the aroma profiles of wines, it was suggested that they can also be considered as part of the “terroir effect” (Figure 1).

But what would be a terroir yeast? Does this suppose that a yeast would be dominant in a wine area and present only in this area, and that this same yeast would be responsible for spontaneous alcoholic fermentation during each vintage? Among the first works performed on the subject, the paper by Vezhinet et al. (1992) [2] is of particular interest. They observed at the level of a large area (Champagne vineyard) that there was not only one strain of *S. cerevisiae* but several strains. Some of these strains were shown to be widely distributed and predominant in the flora involved in must fermentations. The authors suggested that since the strains were widely distributed in the area and because of their remanence over the years, this could express initial evidence for the occurrence of specific native strains, i.e., strains representative of an enological area, namely “terroir” [2]. Later on, using mitochondrial DNA restriction analysis, Gutierrez et al. (1999) [1] studied the number of different strains detected for each vintage over five consecutive years and found that their frequency of occurrence varied from one year to another. A small number of strains were present in consecutive years, but the presence of each one varied as a function of the specific year. Only one strain was present in all the five years studied. For the 1997 vintage, an unusual dominance of non-*Saccharomyces* yeasts in vigorous fermentation was detected; this may explain the abnormal analytical data for the wines of that year. Based on their results, it is not possible to affirm that typical strains of *S. cerevisiae* play an important role in all vinifications. Their results support those of Schutz and Gafner (1994) [15], who also showed that yeast populations differ from one year to another. 

Thus, considering the instability of strains from one year to another and the fact that fermentation is conducted by different strains of *S. cerevisiae* whose frequencies vary from one year to another, these facts do not support the concept of yeast terroir.

However, it may be that yeast terroir is not linked to one yeast strain but that specific native *S. cerevisiae* strains could be associated with a terroir and have an influence on terroir-associated wine characteristics. Furthermore, previous studies did not compare *S. cerevisiae* populations between different geographical locations that, if they were different, could support regional delineations of yeast populations. These two hypotheses have been tested in several studies.

Schuller et al. (2005, 2007) [16,17] reported a large-scale study on the vineyard-associated strains from the Vinho Verde Region in Portugal during three consecutive years. They observed the perennial presence of a strain whose prevalence is reflected in the local microflora. However, this strain was not dominant in each fermentation, showing that the final outcome of fermentation was dependent on the specific composition of the yeast community in the must, which is influenced by many factors. In another study, the authors isolated different *S. cerevisiae* strains from different vineyards in Austria and they concluded, based on Amplified Fragment Length Polymorphism analysis, that the strain clusters obtained correlated well with the geographical distribution of the yeasts [18]. The authors also investigated whether the differences at the genomic level had an impact on the wine volatile profile. They reported that the aroma components’ profile correlated to the geographical origin of the yeast isolates. In a similar but more extensive study, Gayevskiy and Goddard (2012) [19] tested for geographic differences in microbial communities and populations. They were able to demonstrate the existence of geographical differences between grape microbes. Indeed, they showed that different regions of New Zealand contain different genetically distinct and highly related subpopulations of *S. cerevisiae*. However, it should be noted that most of the results were obtained from yeast sampling during a unique vintage, which, as stated above, may not reflect the real ecosystem. This raises another question. How long does it take to consider a strain as a native microorganism of a particular area [20]?

Nevertheless, they concluded that there are region-specific communities and populations of microbes associated with vines and wines. It should be underlined here that the vineyard studied covered a very vast region spanning 350 km, and each vineyard sampled in each region was separated by a distance of 5 km from the others. Another question raised by Vigentini et al. [20] was the following: What can be considered as a terroir? Indeed, in Burgundy in the same village one can find up to 20 different vineyards which are considered to be as many different terroirs, some of which are separated by only a few meters. Could we still stick to the concept of terroir when vineyards are separated by several tens of kilometers? Put differently, where should we place the boundary line when assigning the membership of a strain to a territory [20]?

In another study, Knight et al. (2015) [4] provided the first evidence that there is a positive correlation between microbial relatedness and aroma profiles in wine—in other words, their work supported the idea that local yeasts, unique to each vineyard area, were part of the terroir, contributing to the local flavor of wine. However, the picture seems to be more complex. Indeed, several studies revealed that there are no strains representative of a winery or an area [20,21,22]. By comparing the *S. cerevisiae* biodiversity of two regions in Spain (Priorat and Terra Alta) during three consecutive years, Torija et al. (2001) [21] reported that the yeast population changed from year to year. Furthermore, they observed that some identical strains present in different cellars in the same area were also present in the cellars of different areas. In their study, Vigentini et al. (2015) [20] isolated 270 *S. cerevisiae* strains during three consecutive years in two northern Italian vine-growing territories (six sites in each region). Based on the polymorphism analysis of the interdelta region of all the strains, it appeared that the year of isolation (vintage) proved to be a factor that significantly affected the biodiversity of the yeast species, whereas the geographical site (terroir) was not [20]. In a recent study, the biodiversity of *S. cerevisiae* was studied in 11 Spanish wineries from the Rioja region during 3 or 4 consecutive years [22]. Their conclusion indicated that yeast strains were different each year in each winery, and hardly any common strains were detected between neighboring wineries, which would indicate that there are no representative strains from the winery or the area. In a study conducted in the Sauterne region (France) on three different wine estates, Borlin et al. (2016) [11] observed that high genetic differentiation existed among *S. cerevisiae* populations between the three wine estates. The authors considered that the differences could not be attributed to the geographic distances between the wine estates (less than 10 km). Thus, many studies are contradictory, but one of the reasons could be that some of them were conducted at the country scale [19,23] or at the regional scale [11,20,22]. All these studies focused on *S. cerevisiae*, but many others considered the whole yeast community.

## 3. Terroir Microbial Community

Because of next-generation sequencing (NGS) techniques, microbial populations in the vineyard, grapes, must, and wine have been studied in detail [24,25,26,27]. Some studies using these tools revealed that grape fungal populations were associated with the geographical locations of the vineyards, leading to the concept of microbial terroir. 

In the study of Bokulich et al. (2014) [5], using a high-throughput, short-amplicon sequencing approach, they reported that non-random regional distributions of grape microbiota exist across large geographical scales, meaning that different vineyard regions can be distinguished based on their bacterial and fungal profiles (genus and species). They also presented evidence that grape variety and climatic factors were involved in shaping the microbial pattern, supporting the concept of “microbiome terroir”. Since then, other authors have reported that geographic location is one of the factors shaping the microbial community in the vineyard. Del Portillo et al. (2016) [28] were able to differentiate Priorat vineyards’ region based on their specific bacterial pattern (bacterial phyla). More recently, Drumonde-Neves et al. (2017) [29] revealed qualitative and quantitative variations in the yeast flora composition (species level) between several locations on five islands of the Azores Archipelago. Taylor et al. (2014) [30], and more recently Morrison-Whittle and Goddard (2018) [27], showed that fungal communities in must differ significantly across regional scales (genus level).

The next step was to investigate if these specific regional microbial patterns are in part responsible for wine typicality, and if there is a link between wine chemical composition and the regional associated grape microbial community. Bokulich et al. (2016) [25] succeeded in demonstrating that wine microbiota exhibiting regional patterns correlate with wine chemical composition, suggesting that the grape microbiome may influence terroir.

All these studies agree on the fact that certain regions have “signature” microbial populations. However, some weaknesses of these studies should be stressed. If we look at the study of Bokulich et al. (2014) [5], the authors concluded from their results that regional, site-specific, and grape variety factors shape the fungal and bacterial consortia inhabiting wine-grape surfaces. However, their samples were not grape surfaces but must. One may wonder if must microbial diversity is representative of grape surface. Indeed, in their study, grape must consisted of destemmed, crushed grapes which were subsequently pressed. In these conditions, it is more than likely that winery resident species could have been transferred to the must. Indeed, it is now well accepted that strains involved in spontaneous fermentation originated both partly from the vineyard and the winery [11,31,32,33,34]. Another weakness of these results based on meta-barcoding is their taxonomic resolution, which only goes as far as the genus or species level. Thus, these studies underestimate the biodiversity of the grape microbiome and it may be that a better resolution at the species and even strain level for each species could give a different picture regarding the link between vineyard location and microbial population. Another problem raised by Setati et al. (2012) [6] is that of significant species heterogeneity between samples in the same vineyard. The authors have shown that intra-vineyard variability is a significant factor, and may in some cases be greater than inter-vineyard differences. Finally, amplicon sequencing is not free of pitfalls, and different biases have been described in multiple steps of the process recently reviewed [35].

## 4. The Influence of Anthropogenic Factors on the Grape and Wine Microbial Consortium

Although the definition of terroir encompasses anthropogenic factors, studies have reported that inside a so-called terroir, factors such as vineyard management profoundly affect the microbial consortium, raising the question of the role of this consortium in the characteristics of the “terroir”. Many different strategies of vineyard management exist such as conventional, organic, and biodynamic. In a recent study, Grangeteau et al. compared the impacts of three different phytosanitary treatments by pyrosequencing, namely organic (copper and sulfur fungicides), conventional, and ecophyto (corresponding to dose reduction compared with conventional treatment) treatments, on the biodiversity of fungal populations on grape berries from the same terroir [33]. A decrease in biodiversity (number of genera and Shannon’s index) was measured for three successive vintages for the grapes of the plot protected by an organic treatment compared to other treatments. The impacts of chemical and biological fungicides (based on copper and/or sulfur) on yeast species diversity were also compared by Milanovic et al. (2013) [36]. These authors also observed lower diversity of yeast species and of the *S. cerevisiae* genotype for grape must from the organic vineyard. Other studies pointed out the effect of vineyard management on fungal biodiversity [24,37]. Taking into account that intra-vineyard diversity is important, and that vineyard management greatly influences the grape microbiome, one may wonder if grape yeast biodiversity really plays a role in the terroir concept. In addition to the impact of vineyard management on the grape microbiome, winemaking practices also impact must yeast biodiversity. The addition of selected yeast in must is a common practice in which the commercial *S. cerevisiae* strain dominates the whole alcoholic fermentation process. Must inoculation is currently prevalent worldwide. Although there are no available published figures, most wineries use massive quantities of yeast in the form of active dry yeast. This practice modifies the dynamics of yeast population and the wine sensory character. An interesting study [38] demonstrated that the added *S. cerevisiae* dominated from the middle of the fermentation until the end. They also reported that the addition of a starter limited growth of non-*Saccharomyces* yeast. The sensory profiles obtained for indigenous fermentation versus inoculated fermentation were different. Thus, the addition of selected yeast changes the population dynamic. This was confirmed later in different studies [39,40,41] where the authors showed that early inoculation of must resulted in a lower non-*Saccharomyces* population during alcoholic fermentation and prevalence of the used starter yeast. In these conditions, the contribution of indigenous yeasts to distinctive local wine can be questioned.

Grangeteau et al. [33] reported the strong influence of SO_2_ on populations present during fermentation, and especially the early implantation and domination of the genus *Saccharomyces*. Similarly, it was shown that SO_2_ addition affects wine microbial communities in a dose-dependent manner [42]. Using a metabolomic approach, Grangeteau et al. [33] demonstrated that the composition of wine made from a must with or without sulfite addition leads to two different wines. In another experiment, a series of bottle-aged Chardonnay wines made from the same must, but with different concentrations of SO_2_ added at pressing, were analyzed by ultrahigh resolution mass spectrometry (Fourier transform ion cyclotron resonance (FT-ICR) Mass-Spectrometry and excitation emission matrix fluorescence (EEMF), showing that metabolic fingerprints from FT-ICR-MS data could discriminate wines according to the concentration added to the must [43]. Indeed, sulfite leads to many different changes in wine composition, including reshaping the microbial community in must, as microbes are more or less resistant to sulfite [42,44,45,46]. In these conditions, determining whether the sulfite effect dominates the terroir effect still needs to be investigated.

## 5. The Role of Non-*Saccharomyces* Yeast

As stated above, different studies have shown that different viticultural terroirs harbor distinctive yeast biota [5,26,27,29]. However, in those four studies, yeast was identified at best at the species level [29] or genus level [5,26,27]. Despite the fact that, based on statistics, the data allow highlighting regional microbial patterns from the biological point of view, the description of a microbiome consortium at the genus or species level presents different weaknesses. Indeed, there are many different species and many different strains at the species level within each genus identified [47,48,49]. For example, *Hanseniaspora* spp. have been detected in must in several studies [27,50]. Recently, studies have reported that there is significant variability among important enology parameters at the clonal level when fermentations among several different *Hanseniaspora uvarum* are compared [50]. The same phenomenon has been observed for other non-*Saccharomyces* yeasts such as *Lachancea thermotolerans* [51]. Consequently, it could be that two vineyards can be considered to be the same terroir because they share a very similar yeast consortium at the genus or species level. However, at the yeast strain level these two vineyards may be very different due to the presence of very different yeasts (although belonging to the same species) in each vineyard and which produce different wines.

In the study of Pinto et al. [52], the authors showed that different regions could be discriminated based on the initial must consortium’s characteristics. For example, in that study, the prevalence of *Lachancea* in the Alentejo appellation was reported, whereas that of *Rhodotorula* and *Botryotinia* was shown in the Estremadura appellation. Finally, *Ramularia* and *Hanseniaspora* were the dominant genera in Bairrada, *Rhodotorula* and *Lachancea* in Dão, *Rhodotorula* and *Erysiphe* in Douro, and *Rhodotorula* and *Alternaria* in the Minho appellation. However, the identification was at the genus level, which means that in each genus there could have been many different species and yeast strains. These genera are ubiquitous throughout the world and, as stated above, there are many different species inside each genus and many different clones inside each species. Moreover, what was the contribution of these genera to the organoleptic profiles of the wines? Indeed, while non-*Saccharomyces* are present in must, these species decrease during alcoholic fermentation due to the stressful environment [53]. The population levels of these non-*Saccharomyces* yeasts are highly variable during the course of alcoholic fermentation but, curiously, their impact on the final quality of the wine has been poorly characterized. Indeed, while there is a considerable number of studies showing the benefit of using non-*Saccharomyces* yeasts together with *S. cerevisiae* in co-culture [54], there are very few studies showing the contribution of non-*Saccharomyces* yeasts during indigenous alcoholic fermentation [38,55,56]. Thus, the exact role of non-*Saccharomyces* yeasts present in indigenous fermentation in the final wine composition still requires investigation.

## 6. Cellar Yeast vs. Grape Yeast

**Figure 2:**
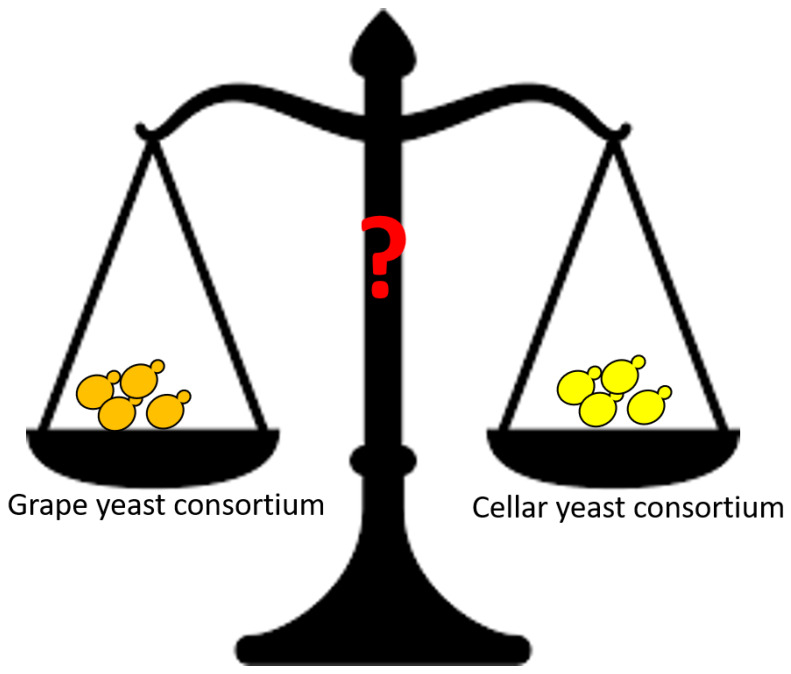
Does Microbial terroir exist? Who is the main player in wine typicality? Terroir yeast from grapevine or cellar yeast?

Another interesting question when speaking about wine yeast terroir is the following: Which yeasts are responsible for alcoholic fermentation, grape or cellar yeasts (Figure 2)? Indeed, it has been reported that *S. cerevisiae* strains may persist in the same winery over consecutive years [2,57], which leads to the concept of “winery effect” [34]. Some of these strains in the winery environment, described as persistent over several years, have been found in grape musts prior to fermentation and observed to become dominant during fermentation to the detriment of strains from the vineyard [31,57]. More recently, it has been shown that other yeast species are natural inhabitants of wineries, such as *Aureobasidium*, *Candida, Cryptococcus*, *Hanseniaspora*, *Metschnikowia*, *Torulaspora*, *Pichia*, *and Williopsis* [10,32,58,59,60]. Using FT-IR spectroscopy to discriminate the isolates of yeasts belonging to the genus *Hanseniaspora* up to the strain level, Grangeteau et al. (2015) [9] demonstrated for the first time the implantation in grape must of two different yeast strains belonging to the genus *Hanseniaspora* present in the winery environment. All these studies revealed that not only yeast from grapes but also from the winery are involved in the transformation process of grape must. The yeasts in the winery environment can thus be present in musts whatever their origin or their terroir. A recent study supported this point of view [61]. The authors suggested the existence of a highly winery-specific “microbial-terroir” contributing significantly to the final product rather than a regional “terroir”.

## 7. Conclusions

While it is quite clear that the microbial community of a vineyard is dependent on geographic location, to my opinion, there is no strong evidence that microbes contribute to the so-called “terroir effect”. As described above, while some studies revealed that grape-associated microbial biogeography is non-randomly associated with regional, varietal, and climatic factors across multiscale viticultural areas, this microbial terroir concept is still the subject of intense debate for the reasons summarized below.

One of the main reasons is the definition of “terroir”; today, this term is used to define every wine-growing region. In these conditions, it is not surprising to see a microbial signature of wine-growing areas located several tens of kilometers from each other. Thus, it remains to be demonstrated that two close terroirs having two different sensorial signatures harbor different microbial profiles and that these specific microbial consortia are in part responsible for different wine typicalities.

Another point that deserves further research is the fact that the results of most studies were obtained from yeast sampling during a single vintage, which may not reflect the real ecosystem. Moreover, the concept of “microbial terroir” is based on differences at the genus or species level. Taking into account the high diversity of each species, the concept should be studied in depth, encompassing a strain-typing level. It should also be underlined that most studies did not include any sensorial analysis. Regional differences were based on the production of volatile organic compounds (VOCs); however, VOC composition does not necessarily lead to different wines from a sensorial point of view. Thus, the influence of microbial signature on wine typicity should include sensorial analysis. 

Another important point that needs further investigation is the impact of non-*Saccharomyces* yeasts on the diversity and concentration of aroma compounds and their influence on the sensorial profile. Indeed, even if a microbial signature could be found, the effect of a species present at concentrations a hundred or a thousand times less than that of the main contributor, i.e., *S. cerevisiae*, remains to be determined.

Thus, there is still a long way to go before closing the debate.
